# Whole-genome sequencing reveals genetic diversity and transmission dynamics of Mycobacterium bovis in South African wildlife

**DOI:** 10.1099/mgen.0.001646

**Published:** 2026-02-10

**Authors:** Abisola Okunola, Johannes Loubser, Giovanni Ghielmetti, Rachiel Gumbo, Elizabeth M. Streicher, Pamela Ncube, Anzaan Dippenaar, Wynand J. Goosen, Ana Marcia Sá Guimarães, Robin M. Warren, Andre G. Loxton, Michele A. Miller, Tanya J. Kerr

**Affiliations:** 1 South African Medical Research Council Centre for Tuberculosis Research; Division of Molecular Biology and Human Genetics, Faculty of Medicine and Health Sciences, Stellenbosch University, Cape Town, South Africa; 2 Section of Veterinary Bacteriology, Institute for Food Safety and Hygiene, Vetsuisse Faculty, University of Zurich, Winterthurerstrasse Zurich, Switzerland; 3 Family Medicine and Population Health, Faculty of Medicine and Health Sciences, University of Antwerp, Antwerp, Belgium; 4 Department of Microbiology and Biochemistry, Faculty of Natural and Agricultural Sciences, University of Free State, Bloemfontein, South Africa; 5 Department of Microbiology, Institute of Biomedical Sciences, University of São Paulo, São Paulo, Brazil

**Keywords:** Greater Kruger Conservation Area, genomic diversity, Hluhluwe–iMfolozi Park, *Mycobacterium bovis*, phylogenetic relationship, South African wildlife, whole-genome sequencing

## Abstract

*Mycobacterium bovis* infection poses a significant threat to the biodiversity and conservation of South African wildlife. Despite this, few studies have explored transmission dynamics within these complex multi-host systems. This study used whole-genome sequencing to investigate the genetic diversity and relatedness of *M. bovis* strains across various wildlife species and regions in South Africa to explore transmission patterns. A total of 112 *M*. *bovis* isolates from 106 individuals representing 12 species underwent short-read sequencing. Two animal-adapted sub-lineages, La1.7.1 (clonal complex Eu2) and La1.8.1 (Eu1), exhibited geographic clustering and notable genomic diversity. Closely related isolates (≤5 SNP differences) were primarily found within single host species, particularly African buffalo (*Syncerus caffer*), indicating intra-species transmission and potential source identification. In contrast, other genetically similar isolates (≤12 SNP differences), collected over 25 years, suggest historical inter-species transmission. Understanding these transmission patterns is essential for developing effective strategies to control the spread of *M. bovis* and protect vulnerable wildlife populations.

**Impact Statement**

Animal tuberculosis (TB), caused by *Mycobacterium bovis*, is a major threat to South African wildlife, yet its transmission dynamics remain poorly understood. This study applies whole-genome sequencing to examine the genetic diversity and relatedness of *M. bovis* strains across multiple wildlife species and regions. By analysing genetic similarity, a method proven effective in tracing TB outbreaks in humans, livestock and wildlife, this research enhances our understanding of disease spread in South African wildlife. The results reveal substantial genetic variation between regions and identify closely related strains that indicate recent transmission events and potential sources. As iconic and endangered species such as the black rhinoceros (*Diceros bicornis*) and African wild dog (*Lycaon pictus*) are vulnerable to infection, these findings are vital for informing targeted management strategies to prevent TB transmission and protect biodiversity.

## Data Summary

The authors confirm that all supporting data, code and protocols have been provided within the article or through supplementary data files. Raw sequencing data for all sequenced isolates have been deposited in the European Nucleotide Archive under project PRJEB75245. Accession numbers are provided in Table S1.

## Introduction

*Mycobacterium bovis* is a member of the *Mycobacterium tuberculosis* complex (MTBC) and one of the causative agents of animal tuberculosis (TB), primarily affecting cattle (*Bos taurus*) [1,4]. However, *M. bovis* has a broad host range and can infect domestic animals, livestock, a wide variety of wildlife species and humans [5,8]. This extensive host range complicates control efforts, particularly at human–livestock–wildlife interfaces in Africa, where underreporting is common due to limited surveillance, diagnostic testing and traditional cattle husbandry practices [7,9,11].

In South Africa, many communal farmers live near game parks, reserves or ranches, where livestock may interact with wildlife [9]. These interactions may facilitate inter-species transmission events [6,11, 12]. Spillover from cattle to African buffaloes (*Syncerus caffer*) has led to this species becoming the principal wildlife maintenance host of *M. bovis* on the continent [13,14]. In South Africa, confirmed *M. bovis* infections have been reported in at least 23 other wildlife species that cohabitate with African buffalo [5,15]. As a result, animal TB is now endemic in several key wildlife reserves, including the Greater Kruger Conservation Area (GKCA), Hluhluwe–iMfolozi Park (HiP) and Madikwe Game Reserve (MGR) [9,16, 17]. These reserves host some of South Africa’s most iconic wildlife species, and the presence of animal TB threatens conservation programmes due to movement restrictions and quarantine measures associated with disease control policies [13,18]. The lack of validated diagnostic TB tests for wildlife further complicates translocation and conservation efforts in TB-affected regions [15].

Historically, epidemiological investigations of animal TB have relied on conventional genotyping methods, such as spacer oligonucleotide typing (spoligotyping), mycobacterial interspersed repetitive unit–variable number tandem repeat and restriction fragment length polymorphism to characterize *M. bovis* and infer transmission patterns [11,12, 19,21]. However, these methods offer limited resolution and may overestimate transmission, potentially leading to misinterpretation of epidemiological data [2,22]. For instance, spoligotyping is inadequate for establishing links at livestock–wildlife or animal–environment interfaces due to the slow evolutionary rate of the targeted direct repeat region, which can result in over-clustering of isolates [18,23, 24]. While variable number tandem repeat (VNTR) analysis improves detection of genetic diversity and transmission inference, it still lacks the resolution of more advanced techniques [2,20].

Whole-genome sequencing (WGS) has emerged as a powerful tool for investigating the molecular epidemiology of animal TB. Due to the generally low mutation rate observed with MTBC, WGS-based genotyping typically uses low SNP thresholds (five SNPs) to determine epidemiologically linked cases [25]. While WGS provides finer resolution for clustering compared to traditional genotyping, determining who infected whom remains challenging when relying solely on genomic data [25,29]. To date, only three studies in South Africa have applied WGS to assess *M. bovis* diversity in wildlife, each using relatively small datasets [30,32]. The study by Dippenaar *et al*. [30] included 15 isolates from several species (African buffalo, African lion, leopard, greater kudu and Chacma baboon), while the study by Meiring *et al*. [31] included 5 isolates from 4 African wild dogs, and the study by Roos *et al*. [32] included 8 isolates from common warthog. Therefore, this study aimed to investigate the genetic diversity of *M. bovis* in South African wildlife using WGS and to explore its utility in understanding TB epidemiology within complex multi-host ecosystems.

## Methods

### *M. bovis* isolation, identification and spoligotyping

**Fig. 1.**
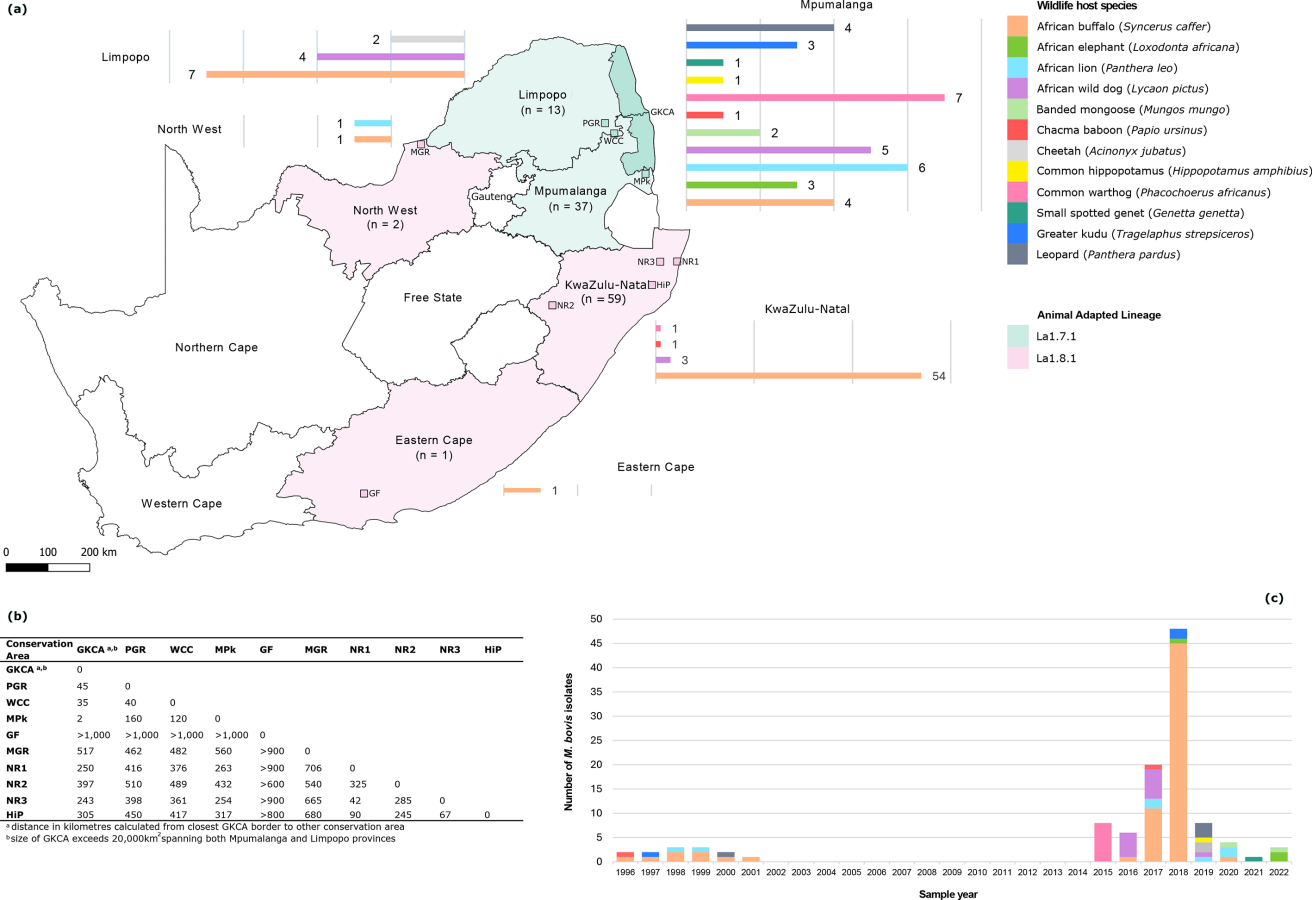
(**a**) Map of South Africa showing the number and origin of the *M. bovis* isolates by geographic region (province) and wildlife host. Shading of geographic regions (provinces) and conservation areas was used to indicate the geographic distribution of animal-adapted sub-lineages. Conservation areas: MGR, GKCA, MPk, PGR, WCC, HiP, NR 1–3 and GF. (**b**) Distance matrix showing pairwise distances (in kilometres) between conservation areas. (**c**) Number of *M. bovis* isolates collected per year (1996–2022).

*M. bovis* isolates were obtained from previously cultured, but unsequenced, wildlife samples collected across five South African provinces between 2016 and 2022 (Fig. 1a, c). The original post-mortem tissue specimens were opportunistically collected during veterinary procedures, with ethical approval. Samples originated from free-ranging wildlife in nine locations: the GKCA, HiP, as well as smaller populations in a private game reserve (PGR), private game farm (GF), Marloth Park (MPk), MGR and three nature reserves (NR1-3). In addition, *M. bovis* isolates from four captive animals at a wildlife conservation centre (WCC) were included. Estimated distances between conservation areas are shown in Fig. 1b. Distance in kilometres was calculated from the closest GKCA border to other conservation areas. The size of GKCA exceeds 20,000 km^2^, across Mpumalanga and Limpopo provinces. None of the locations had contiguous borders or shared wildlife corridors (Fig. 1a). Tissue samples were processed for mycobacterial culture as described by Kerr *et al*. [33]. Positive cultures were speciated using genomic regions of difference (RD) PCR [34] and spoligotyping [35]. Confirmed *M. bovis* isolates were stored at −80 °C in glycerol peptone medium on glass beads.

For reactivation, bead stocks were inoculated into Mycobacteria Growth Indicator Tubes (MGIT^™^ Becton Dickinson, Franklin Lakes, NJ, USA) with 800 µl of BD BACTEC^™^ MGIT^™^ 960 Supplement and incubated in a BACTEC^™^ MGIT^™^ 960 Mycobacterial Detection System for at least 56 days (all Becton Dickinson). Positive cultures were sub-cultured by transferring 1 ml of liquid MGIT culture suspension into 5 ml of freshly prepared 7H9 broth, supplemented with OADC (Becton Dickinson) and incubated at 37 ℃ for 14 days. Cultures were then heat-inactivated at 80 °C for 1 h and aliquoted for transport to the US Department of Agriculture’s National Veterinary Services Laboratories (NVSL) (Ames, IA, USA) for WGS.

### WGS and sequence analyses

At the NVSL, genomic DNA was extracted using the KingFisher Flex System (Thermo Fisher, Waltham, MA, USA) and MagMax CORE Kit (Thermo Fisher), according to the manufacturer’s instructions [36]. Libraries were prepared using the Nextera XT Kit (Illumina, Inc., San Diego, CA, USA) and sequenced on an Illumina MiSeq platform using 2×250 bp paired-end chemistry. A minimum of 20 µl of DNA at ≥5 ng µl^−1^ was required. Raw reads were deposited in the European Nucleotide Archive (ENA) (Project Accession: PRJEB75245; Table S1, available in the online Supplementary Material). In addition to the 78 new sequences, 34 previously published *M. bovis* sequences from South African wildlife were retrieved from ENA (project accession numbers PRJEB18668 [30], PRJEB39449 [31], PRJEB27859 [32] and PRJEB63553 [36]), resulting in a final dataset of 112 sequences (Tables S1 and S2).

Newly sequenced and publicly acquired sequences were trimmed and adapters removed with Trimmomatic v0.38, sliding window 4 : 20 using a minimum phred score of 20 [37] before screening with Kraken2 (16GB-capped standard database). Trimmed reads were assessed for quality using FastQC v0.11.9 [38], and the following quality parameters were applied: genome coverage after trimming ≥30×, average read length ≥75 bp, GC content of ~65 mol % without multiple peaks and the absence of bacterial contaminants according to Kraken2 [39]. Bracken was used to summarize the output from Kraken2 analysis [40], and the top 20 identified species from the Bracken output were used to determine if the sample was contaminated with other micro-organisms. The trimmed and filtered reads were then used as input for bovisanalyzer (https://github.com/avantonder/bovisanalyzer), using BWA-MEM to align to *M. bovis* AF2122/97, followed by sorting and indexing of alignments using SAMtools v1.15 [41,42]. Following read mapping, sequences were retained if ≥95 % of the reads mapped to the reference genome, with ≥95 % coverage of the reference genome. Duplicate read marking was performed by Picard v2.27.4 [43] and variants called and filtered using BCFtools v1.15.1 [44]. Filtered VCF files were converted to consensus FASTA files using BCFtools (http://samtools.github.io/bcftools/howtos/publications.html), and each FASTA file was masked with zero coverage, low coverage and poorly mapped regions (https://github.com/avantonder/bovisanalyzer/blob/main/assets/DataDrivenMerge20.bed), including repetitive PE/PPE gene families, using BCFtools and BEDtools v2.3.0. The percentage of the reference mapped in each FASTA file was calculated using seqtk v1.0 (https://github.com/lh3/seqtk). Consensus FASTA files from all samples were aligned with an in-house Python script (File S1) before extracting variants using SNP-sites v2.5.1 [45]. Lastly, the original trimmed and filtered reads were also analysed by TB-Profiler v6.0.0 to determine lineage, and the resulting VCF files were screened for mixed infections using a Gaussian mixture model tool, GMM4TB [46].

### Phylogenetic reconstruction, pairwise SNP distance and minimum spanning tree

Following quality control, mapping and variant calling of an initial 136 newly sequenced isolates, a total of 78 newly sequenced and 34 previously published *M. bovis* genomes from South African wildlife were included in the phylogenetic analysis. Additionally, 198 publicly available *M. bovis* sequences from European cattle, red deer and wild boar that met quality control criteria were retrieved from ENA repositories PRJNA946560 [23] and PRJEB9025 [26] (Table S3). Three distantly related *Mycobacterium caprae* isolates (*n*=3; ERR1462581, ERR1462585 and ERR1462625) were used as an outgroup. The concatenated sequences of these isolates (112 wildlife-derived, 198 publicly acquired sequences and 3 outgroup isolates), comprising 5,717 high-confidence variable sites, were used to construct a maximum likelihood phylogeny with IQ-TREE [47]. The analysis employed an automatic nucleic acid substitution model selection using ModelFinder, which identified GTR+F+ASC+R2 as the best-fit model, incorporating ascertainment bias correction. Node support was assessed using 1,000 ultrafast bootstrap replicates, and nodes with bootstrap support ≥75 % were considered robust. The resulting phylogenetic tree was visualized using the Interactive Tree of Life (iTOL) web-based tool [48].

A SNP distance matrix was generated from the aligned FASTA sequences, excluding the outgroup strains (*n*=3) and European cattle and wildlife sequences (*n*=198). Genetic relatedness according to pairwise SNP distance was interpreted as follows: isolates differing by ≤5 SNPs were considered closely related and potentially epidemiologically linked, those differing by up to 12 SNPs were considered genetically similar and isolates with >12 SNP differences were not considered genetically related, as described previously [25,49, 50].

To further explore genetic relationships, a minimum spanning tree (MST) was constructed using the web-based tool PHYLOViZ (https://online.phyloviz.net/index). The MST was constructed using the same FASTA alignment used for the SNP matrix [51], and links were visualized as absolute distances.

## Results

### South African wildlife host species, temporal and geographic distribution of *M. bovis* isolates

The final *M. bovis* WGS dataset (*n*=112) included isolates from 106 unique individual animals across 12 wildlife species (Tables S1 and S2). These comprised African buffalo (*n*=65), African elephant (*n*=2), African lion (*n*=7), African wild dog (*n*=11), banded mongoose (*n*=2), Chacma baboon (*n*=2), cheetah (*n*=1), common hippopotamus (*n*=1), small spotted genet (*n*=1), greater kudu (*n*=2), leopard (*n*=4) and common warthog (*n*=8). Multiple tissue samples from six individuals yielded two *M. bovis* isolates per animal (Table S1). In all six cases, the WGS results from different tissues were identical (i.e. pairwise SNP distance=0). Importantly, no mixed infections were detected.

Isolates were cultured from samples collected between 1996 and 2022, with most (87.5%, *n*=98) collected between 2015 and 2022 (Fig. 1c). Samples originated from five South African provinces: Mpumalanga (*n*=37), Eastern Cape (*n*=1), North West (*n*=2), Limpopo (*n*=13) and KwaZulu-Natal (KZN) (*n*=59) (Fig. 1a). The majority (79%) of samples were from *M. bovis* endemic populations in the GKCA (*n*=33) and HiP (*n*=55). Metadata for all isolates are provided in Table S1.

### *M. bovis* spoligotyping

Spoligotyping and RD PCR confirmed the identity of new isolates as *M. bovis*, based on the absence of spacers 3, 9, 16 and 39–43 in the spoligotype patterns and the characteristic RD deletion profile. A total of ten distinct spoligotypes were identified among the South African wildlife isolates, all of which are registered in the international *M. bovis* spoligotype database (Table S4). The three most common spoligotypes were SB0121 (42/112; 37.5%), SB0130 (38/112; 33.9%) and SB1474 (18/112; 16.1%). Spoligotype SB0121 was the most geographically widespread, detected in 12 wildlife species across Mpumalanga and Limpopo provinces (Table 1). Spoligotype SB0130 was predominantly found in African buffalo from KZN (35/38 isolates), with the remaining three isolates originating from two African wild dogs (also from KZN) and one African lion from North West province. Spoligotype SB1474 was exclusively found in African buffalo and appeared to be geographically restricted to KZN. The temporal distribution of the major spoligotypes was similar; SB0121 and SB0130 were detected over extended periods (1996–2022 and 1997–2018, respectively), while SB1474 was only identified in 2018 (Table 1).

**Table 1.** Summary of *M. bovis* spoligotypes isolated from South African wildlife species, including animal-adapted sub-lineage, clonal complex, geographic distribution (province and conservation area), number of isolates, wildlife host species affected and sample collection period

Spoligotype	Animal-adapted sub-lineage	Clonal complex	Province	Conservation area	No. of isolates	Wildlife host species affected	Sample collection period
SB0121	La1.7.1	Eu2	Limpopo, Mpumalanga	GKCA, MPk, WCC, PGR	42	12 host species*	1996–2022
SB1195	La1.7.1	Eu2	Limpopo	PGR	3	African buffalo	2017
SB1275	La1.7.1	Eu2	Mpumalanga	MPk	3	Common warthog	2015
SB1947	La1.7.1	Eu2	Mpumalanga	GKCA	1	African lion	2017
SB2723	La1.7.1	Eu2	Mpumalanga	MPk	1	Banded mongoose	2020
SB0130	La1.8.1	Eu1	KwaZulu-Natal, North West	HiP, NR1, MGR	38	African buffalo, African lion, African wild dog	1997–2018
SB0131	La1.8.1	Eu1	Eastern Cape	GF	1	African buffalo	2020
SB0140	La1.8.1	Eu1	KwaZulu-Natal, North West	MGR, NR2, NR3	4	African buffalo, chacma baboon, common warthog	1999–2017
SB1474	La1.8.1	Eu1	KwaZulu-Natal	HiP	18	African buffalo	2018
SB2681	La1.8.1	Eu1	KwaZulu-Natal	HiP	1	African wild dog	2016

*Host species included African buffalo, cheetah, African wild dog, greater kudu, leopard, African lion, African elephant, small spotted genet, banded mongoose, common hippopotamus, Chacma baboon and common warthog.

### WGS-based *M. bovis* lineage identification, distribution of spoligotypes and phylogenetic structure

**Fig. 2.**
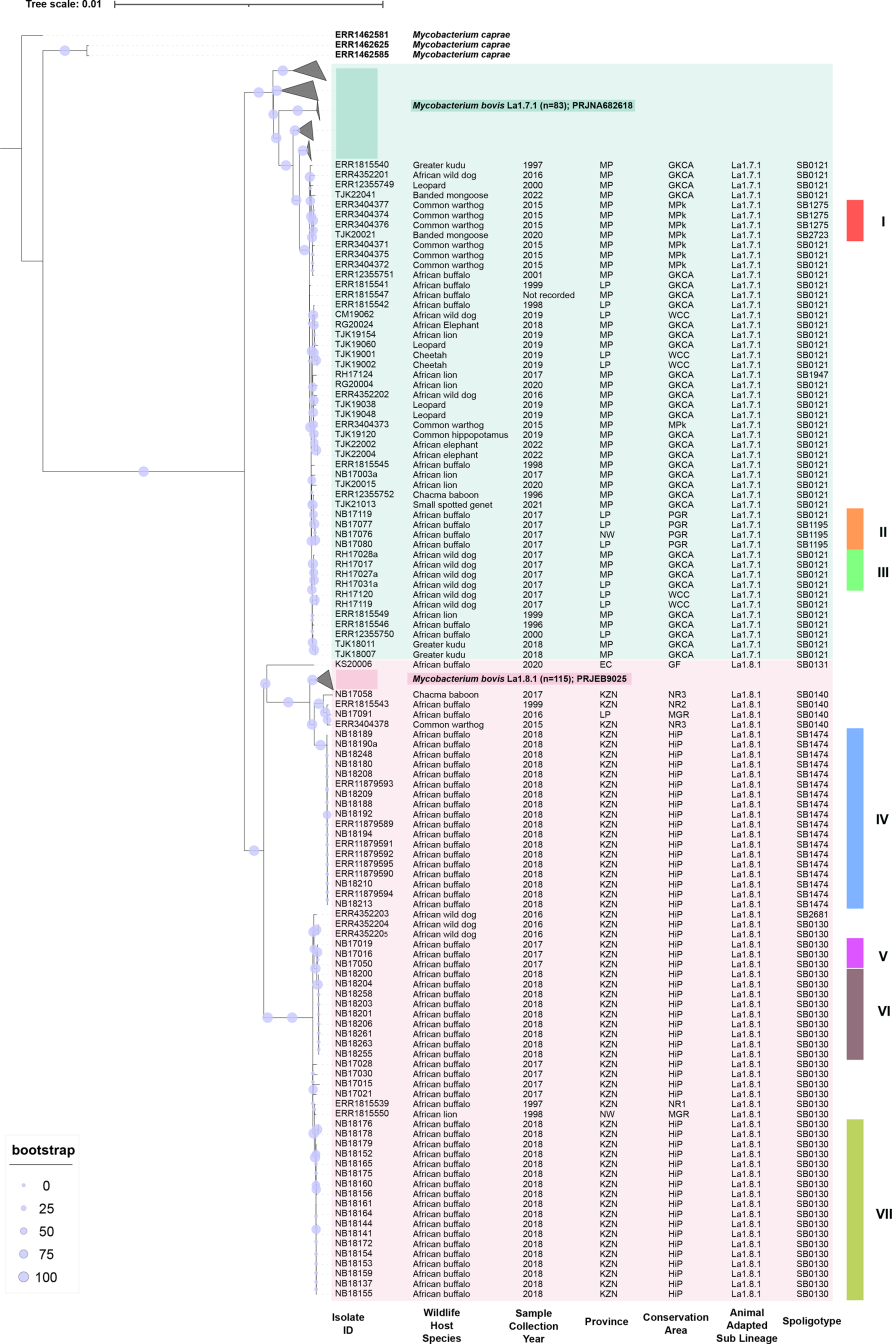
Maximum likelihood tree of 112 *M. bovis* sequences isolated from South African wildlife; 198 publicly available *M. bovis* sequences from European cattle, red deer and wild boar; and 3 distantly related *M. caprae* isolates, which were used as an outgroup. Maximum likelihood bootstrap values (1,000 iterations) are indicated (blue circles). The tree is rooted with *M. caprae*. Each sequence is designated by a unique sequence name comprising ENA accession number (published sequences, Tables S1 and S3) or isolate ID (new sequences generated from this study), wildlife host species, sample collection year, sampling location (province and conservation area), animal-adapted lineage and spoligotype. Shading was used to indicate animal-adapted sub-lineages. Close genetically related clusters (when isolates differed by ≤5 SNPs) are indicated with roman numerals. Province: Eastern Cape (EC), KwaZulu-Natal (KZN), Limpopo (LP), Mpumalanga (MP) and North West (NW).

The phylogenetic relationships of the 112 *M*. *bovis* WGS, along with the distantly related outgroup isolates (*M. caprae*) and European *M. bovis* sequences, are shown in Fig. 2. WGS of *M. bovis* isolates from South African wildlife revealed a single major clade comprising monophyletic sequences that shared a common ancestor (Fig. 2). These sequences were divided into 2 sub-lineages: La1.7.1 (clonal complex Eu2), which included 50 isolates from Limpopo and Mpumalanga, and La1.8.1 (clonal complex Eu1), containing 62 isolates from KZN, Eastern Cape and North West provinces. Isolates from multiple host species were present in both sub-lineages; however, African buffalo were the predominant hosts in La1.8.1.

Spoligotype patterns clustered by geographical region (Fig. 2). Within La1.7.1, five spoligotypes were identified in wildlife: SB0121, SB1275, SB2723, SB1947 and SB1195. The majority (*n*=42/50) were SB0121, detected across 12 wildlife species in the GKCA (spanning Limpopo and Mpumalanga) and surrounding areas, including MPk (Mpumalanga), a PGR (Limpopo) and a WCC (Limpopo) housing a captive cheetah and 3 African wild dogs. These isolates spanned a 26-year period (1996–2022). The remaining four spoligotypes only differed by a maximum of three spacers from the ancestral SB0121 strain. In some cases, different spoligotypes were isolated from the same host species and location, for example, SB0121 and SB1275 (differing by a single deletion) were isolated from common warthogs in MPk (Mpumalanga) in 2015 [32]. Similarly, there were three African buffalo in a PGR (Limpopo) that were typed as SB1195, which differed by only one deletion from the ancestral SB0121 strain, which was also present in an additional African buffalo in this herd.

Among La1.8.1 wildlife isolates, five spoligotypes were also observed: SB0130, SB0131, SB0140, SB1474 and SB2681 (Fig. 2). Unlike La1.7.1, this sub-lineage exhibited lower host diversity, with SB1474 and SB0130 predominantly found in African buffalo from HiP (KZN) and SB0131 in a single African buffalo from a private game farm in the Eastern Cape (GF, 2020). Spoligotype SB0140 occurred in three different host species, a Chacma baboon and a common warthog (NR3, KZN), and two African buffalo from different locations (NR2, KZN and MGR, North West) (Tables 1 and S1). Spoligotype SB2681 – distinguished from SB0130 by a single unique deletion – was identified in one African wild dog (HiP, KZN), while two other African wild dogs sampled in the same location and year were typed as SB0130.

The La1.7.1 clusters exhibited shorter branch lengths compared to those of La1.8.1, indicating lower overall genetic divergence among isolates (range of SNP differences within sub-lineages: 0–49 and 0–386, respectively, Table 2). This pattern persisted despite similar sampling periods (1996–2022 for La1.7.1 and 1999–2018 for La1.8.1). In contrast, the three well-defined and genetically distinct clusters of La1.8.1 exhibited longer branch lengths, consistent with more extensive diversification (Fig. 2).

**Table 2.** Summary statistics of pairwise SNP distances between WGS of *M. bovis* isolated from South African wildlife

Pairwise SNP comparison	No. of isolates (n)	Minimum pairwise SNP distance	Maximum pairwise SNP distance	Mean pairwise SNP distance	Standard deviation
Total	112	0	498	277	195
La1.7.1	50	0	49	26.5	10.4
La1.8.1	62	0	386	177.6	167.8
Cluster I	4	0	2	0.6	0.7
Cluster II	4	0	1	0.3	0.5
Cluster III	4	0	4	1.2	1.5
Cluster IV	18	0	0	0	0
Cluster V	3	0	0	0	0
Cluster VI	9	0	0	0	0
Cluster VII	18	0	1	0.1	0.3

### Epidemiological investigations based on *M. bovis* genetic relatedness

Pairwise SNP distances among the 112 *M*. *bovis* WGS ranged from 0 to 498 SNPs (Tables 2 and S5). Within the province, SNP differences were also variable. For example, isolates from Limpopo and Mpumalanga combined varied by 0 to 49 SNPs, in contrast to isolates from KZN, which ranged from 0 to 386 SNPs and the two isolates from North West province, which differed by 363 SNPs. Within sub-lineages, La1.7.1 isolates had a lower mean SNP distance (26.5 SNPs) compared to La1.8.1 (mean 177.6 SNPs), as shown in Table 2.

Within sub-lineages, multiple spoke-and-wheel cluster patterns of *M. bovis* WGS emerged, consistent with clonal expansion. Seven distinct clusters of closely related isolates (≤5 SNP differences) were identified (Table 3). Sub-lineage La1.7.1 clusters (I–III) were found in different geographic locations in Limpopo and Mpumalanga provinces, whereas the four La1.8.1 clusters (IV–VII) occurred in the same conservation area (HiP, KZN). Within each cluster, mean pairwise SNP differences were <2 (Table 2). As expected, six clusters of closely related sequences were isolated from single host species: five from African buffalo herds and one cluster among African wild dogs (Table 3). Cluster I was the only group of WGS from multiple species – three common warthogs and one banded mongoose from MPk (Mpumalanga), sampled 5 years apart.

**Table 3.** Clusters of closely related isolates (≤5 SNP differences) based on WGS of *M. bovis* isolated from South African wildlife

Cluster ID	Wildlife host species (*n*=**no.** of individuals)	Conservation area	Sample collection year	Isolate ID
I	Common warthog (*n*=3)	MPk	2015	ERR3404374
				ERR3404377
				ERR3404376
	Banded mongoose (*n*=1)		2020	TJK20021
II	African buffalo (*n*=3 animals; 4 isolates)	PGR	2017	NB17119
				NB17077
				NB17080
				NB17076
III	African wild dogs (*n*=4)	GKCA	2017	RH17028a
				RH17017
				RH17031a
				RH17027a
IV	African buffalo (*n*=17 animals; 18 isolates)	HiP	2018	NB18209
				NB18208
				ERR11879593
				NB18180
				NB18248
				NB18194
				ERR11879591
				ERR11879590
				NB18189
				ERR11879595
				ERR11879589
				NB18188
				NB18192
				ERR11879592
				NB18210
				ERR11879594
				NB18213
				NB18190a
V	African buffalo (*n*=3)	HiP	2017	NB17019
				NB17050
				NB17016
VI	African buffalo (*n*=9)	HiP	2018	NB18201
				NB18258
				NB18200
				NB18203
				NB18204
				NB18206
				NB18261
				NB18263
				NB18255
VII	African buffalo (*n*=18)	HiP	2018	NB18178
				NB18176
				NB18179
				NB18175
				NB18160
				NB18156
				NB18164
				NB18161
				NB18152
				NB18144
				NB18141
				NB18165
				NB18153
				NB18155
				NB18172
				NB18159
				NB18154
				NB18137

**Fig. 3.**
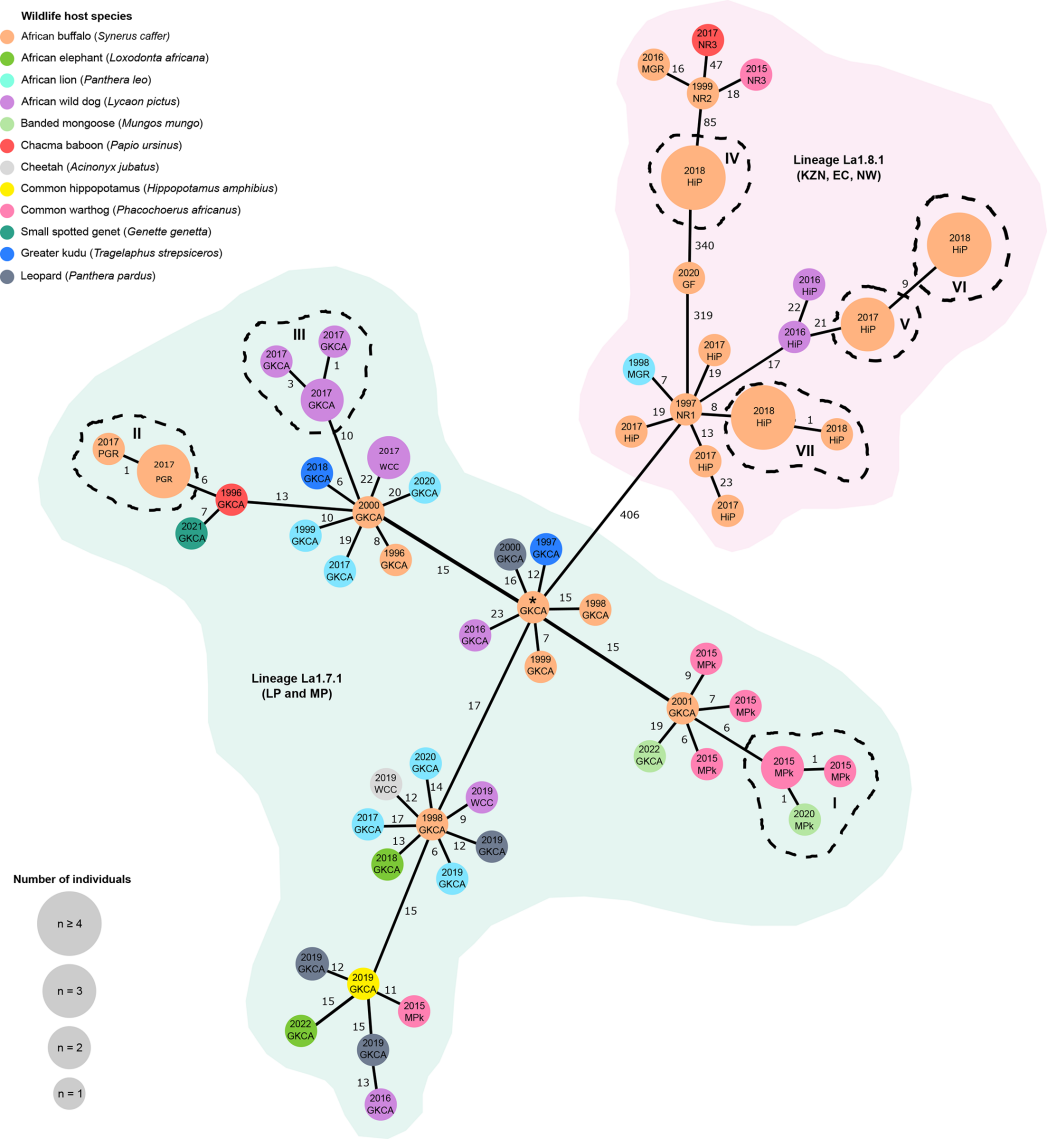
MST based on SNP analysis of 112 *M. bovis* sequences isolated from South African wildlife. Nodes are labelled with the sample collection year and the conservation area where the sample was collected. Nodes are colour-coded according to wildlife host species. Isolates that differ by 0 SNPs form part of the same node. Node sizes are scaled according to the number of individuals represented by that node. Branches connect the nodes that were visualized as an absolute distance. Numbers on branches connecting the nodes represent the number of segregating SNP sites between a pair of nodes. Close genetically related clusters of *M. bovis* sequences (isolates differed by ≤5 SNPs) are represented by dashed lines (----) with roman numeral (I–VII). Shading was used to indicate animal-adapted sub-lineages and geographic locations (provinces). Province: Eastern Cape (EC), KwaZulu-Natal (KZN), Limpopo (LP), Mpumalanga (MP) and North West (NW).

Applying a broader threshold (≤12 SNP differences) to identify genetically similar isolates revealed additional clusters with potential epidemiological links. Although clusters I–III were genetically distinct from one another and occurred in three different conservation areas, the closest *M. bovis* sequences for all three came from historical GKCA (spanning Limpopo and Mpumalanga) isolates (Fig. 3). Cluster I sequences from MPk (isolated in 2015 and 2020) were genetically similar to a 2001 GKCA African buffalo isolate (MPk and GKCA~2 km apart, Fig. 1b). Cluster II African buffalo isolates from a private herd (PGR, Limpopo) differed by six SNPs from a Chacma baboon sampled in GKCA in 1996 (~45 km apart). Similarly, the African wild dog cluster III (GKCA, 2017) varied by ten SNPs from a GKCA African buffalo tested in 2000.

Interestingly, the four sequence clusters (IV–VII) from HiP (KZN) showed variable genetic relatedness, despite being from relatively larger sample sizes, the same host (African buffalo) and a short temporal window (2017–2018). Within-cluster variability was minimal, with mean SNP differences of 0 for clusters IV–VI and 0.1 SNPs in cluster VII (Table 2). Sequences in clusters V and VI differed by only nine SNPs. However, there were 46 SNP differences between clusters V and VII, with cluster IV being the most genetically distinct, differing by >300 SNPs from other HiP African buffalo clusters (Table S5).

Several epidemiological links were evident between isolates from different conservation areas (Fig. 3). An African buffalo from NR1 (KZN, 1997) was genetically similar (nine SNPs) to cluster VII African buffalo from HiP (KZN, 2018), consistent with sequence evolution and historical African buffalo movement between regions (~90 km apart, Fig. 1b). A comparable link was observed between isolates from a captive cheetah and African wild dog from WCC (Limpopo, 2019) and a GKCA African buffalo (1998), differing by 12 and 9 SNPs, respectively. These two conservation areas are ~35 km apart. Similarly, a GKCA African buffalo isolate (2001) was genetically similar (six SNPs) to multiple common warthog isolates (cluster I) from MPk (Mpumalanga, 2015), suggesting movement of infected animals between locations (~2 km apart). However, an African lion isolated from MGR (1998) differed by only 7 SNPs from an African buffalo in NR1 (1997), despite originating from different provinces ~706 km apart (Figs 1b and 3).

In GKCA, inter-species epidemiological links were observed (Fig. 3). Cluster III African wild dog isolates (2017) differed by 10 SNPs from an African buffalo sequence (2000). This African buffalo sequence also showed genetic similarity (10 and 6 SNP differences) to that of an African lion (1999) and a greater kudu (2018), respectively. Notably, GKCA isolates from species unlikely to have direct contact with the African buffalo were genetically linked. For example, a Chacma baboon (1996) differed by only 7 SNPs from an African elephant sequence (2021).

Conversely, large SNP differences were occasionally observed among isolates from the same location: an African lion (1998) and an African buffalo (2016) from MGR differed by 363 SNPs, suggesting distinct infection sources (Table S5). Similarly, *M. bovis* WGS from multiple species in GKCA showed ≥12 SNP differences, which included samples from common hippopotamus, leopard, African elephant and African buffalo (Fig. 3).

## Discussion

WGS has become an essential tool for investigating the epidemiology and evolution of *M. bovis* in animal populations [52,55]. In the context of *M. bovis,* WGS enables the identification of fine-scale genetic differences that are not detectable by traditional genotyping methods such as spoligotyping or VNTR analysis [2]. This study represents one of the most comprehensive genomic investigations of *M. bovis* in African wildlife to date, by analysing 112 isolates from 12 wildlife species across 5 South African provinces. The majority of *M. bovis* WGS were from African buffalo, which are the primary wildlife maintenance host in South Africa [14,17]. This is supported by the predominance of African buffalo sequences at the central nodes in the MST and spatial-temporal clustering of closely related isolates. Although the greater kudu has also been identified as a maintenance host, the small sample size precludes its representation in this study [15,17]. Genetically similar sequences from carnivore samples indicate that spillover may be occurring through shared *M. bovis*-infected prey. Other herbivore species, such as the common warthog, are likely dead-end hosts, becoming infected indirectly through *M. bovis* environmental contamination [32]. These findings underscore the genetic diversity and complex transmission dynamics of *M. bovis* in multi-host ecosystems.

Two major animal-adapted sub-lineages, La1.7.1 (clonal complex Eu2) and La1.8.1 (Eu1), were identified, with clear geographic segregation. On the African continent, La1.7.1 has been limited to South Africa and La1.8.1 to South Africa and Tanzania [55]. In this study, La1.7.1 was found exclusively in Limpopo and Mpumalanga provinces (distances between GKCA, PGR, WCC and MPk were 1–160 km apart), while La1.8.1 was only found in KZN (HiP, NR1, NR2 and NR3 were 42–325 km apart) and in the Eastern Cape (GF) and North West (MGR) provinces, which were >500 km from KZN. Distances between conservation areas in Mpumalanga/Limpopo and KZN, which represented isolates from La1.7.1 and La1.8.1, ranged between 243 and 510 km. This geographic clustering aligns with previous studies suggesting *M. bovis* lineages are more strongly associated with location than host species [54,55].

The presence of European-derived clonal complexes in South Africa likely reflects historic introductions via imported cattle, especially from Britain, with the first report of cattle TB in 1880 in South Africa [15,56,59]. This is supported by the phylogenetic relationships of publicly acquired WGS from European cattle and wildlife with the South African wildlife *M. bovis* isolates from this study. Spillover into wildlife populations was first detected in 1928 in greater kudu and common duiker in the Eastern Cape [60]. Wildlife TB in HiP (KZN) was discovered in a black rhinoceros in 1970, followed by African buffalo in the early 1990s [9,19]. Although an impala was the first diagnosed case in the GKCA in 1967, it was not until 1990 that TB in African buffalo was found in southern KNP [14,61].

Historically, the genetic variability of *M. bovis* isolates in South Africa has been assessed primarily through spoligotyping and VNTR analysis [12,18, 20]. In this study, spoligotyping revealed 10 distinct patterns, with SB0121 and SB0130 being the most prevalent. However, WGS demonstrated that spoligotyping alone is insufficient for resolving transmission dynamics, as isolates with identical spoligotypes often exhibited substantial genomic divergence. This limitation has been noted in other studies, where spoligotyping overestimates relatedness due to its reliance on a slowly evolving genomic region [18,23, 32]. In contrast, WGS provided high-resolution insights into the genetic structure of *M. bovis* populations in wildlife, revealing potential recent and historical transmission events. Unfortunately, investigation of transmission at livestock-wildlife interfaces was constrained by the absence of *M. bovis* genomes from livestock in South Africa. However, future research should incorporate *M. bovis* WGS from all susceptible hosts in South Africa to improve understanding of TB epidemiology.

Seven clusters of closely related isolates (≤5 SNPs) were identified, primarily within African buffalo populations. This supports the role of African buffalo as a maintenance host for *M. bovis* in South Africa, consistent with previous findings [15,17]. The low SNP diversity within these clusters suggests ongoing intra-species transmission, likely facilitated by the social behaviour and herd structure of African buffalo [14]. However, the presence of genetically distinct strains within the same conservation area (e.g. HiP) indicates multiple introductions and independent transmission chains, as previously reported by Hlokwe *et al.* [19].

Considering that *M. bovis* has a slow evolutionary rate and most isolates of La1.8.1 were collected in recent years, rapid local expansion leading to genetic diversity is unlikely [3,55]. Instead, these differences probably reflect distinct *M. bovis* introduction histories, with La1.8.1 clade representing at least two independent introductions of already-divergent strains into the region, whereas La1.7.1 clade may have originated from a single or fewer introductions of more closely related isolates. The La1.7.1 wildlife sequences appear to share a common ancestor with strains from the Iberian Peninsula (Eu2 clonal complex) [23], while those belonging to La1.8.1 shared a distant common ancestor with strains (Eu1) from the UK [26]. The presence of separate La1.8.1 clusters from African buffaloes in HiP (collected in 2017–2018) demonstrates the concurrent circulation of genetically distinct strains within a single host population, indicating that the observed genetic diversity of La1.8.1 is not explained by geographic or host-associated separation. The near-clonal distribution of La1.8.1 strains is also visible from the publicly acquired UK isolates [26].

Evidence of epidemiological links between species was also observed. For example, a single SNP difference was present between a banded mongoose and a common warthog from MPk (Mpumalanga), although they were sampled 5 years apart. These findings suggest transmission may occur through shared burrows or contaminated grazing areas, analogous to TB dynamics in European badgers [62]. The detection of genetically similar isolates in species sampled years apart further supports the role of potential environmental reservoirs or indirect transmission routes [24,63].

Although a SNP threshold of ≤5 has been used to infer recent transmission of MTBC strains [64], studies emphasize that the appropriate threshold should be context-specific, depending on the evolutionary rate of the pathogen and temporal scale of the study [65]. In this study, the extended time span between sample collection for many of the isolates (excluding HiP African buffalo) limited the ability to confidently assign isolates to recent transmission clusters. However, applying a less stringent threshold of ≤12 SNPs enabled the identification of additional genetically similar isolates, which may reflect historical transmission events or movement-associated spread. In this study, *M. bovis* genetic similarity was identified between African buffalo and African lion, as well as between African buffalo and African wild dogs. These findings support the hypothesis that carnivores often act as spillover hosts [17,66]. However, more recent research has demonstrated that African lions and African wild dogs can shed viable *M. bovis* in respiratory secretions, suggesting the potential for intra-species transmission among carnivores [67,68]. Notably, *M. bovis* genetic similarity was also observed among herbivore species – such as African buffalo, common warthog, African elephant, greater kudu and common hippopotamus in the GKCA, despite limited opportunities for direct contact. This raises the possibility of indirect transmission through shared environmental resources, such as waterholes or grazing areas [24,63]. The detection of mixed infections is important both for bioinformatic analyses, to ensure accurate SNP calling, and epidemiologically, to better understand infection pressure on these animals. However, the sampling and culturing approach was not optimal for detecting mixed infections, as in most cases only a single isolate per animal was sequenced, with the exception of six individuals. Investigating within-host diversification would require sampling multiple anatomical sites per animal and applying less stringent cut-offs in the bioinformatic pipeline. Because of the limited geographical diversity of our dataset, the detection of highly divergent *M. bovis* strains was unlikely. As a result, differentiating within-host microevolution from true mixed infections caused by closely related strains remains challenging.

Several genetically similar isolates were found in geographically distant locations, suggesting that translocation of wildlife, through auctions, private sales or conservation initiatives, may contribute to the dissemination of *M. bovis* [16,19]. Transport of infected livestock, especially communal cattle, may also contribute to the undetected spread of these strains [9,69]. Although cattle and African buffalo are currently subject to mandatory TB testing before movement [9], there are critical gaps in surveillance and biosecurity protocols in South Africa. The high prevalence of *M. bovis* in African buffalo in GKCA, along with the lack of culling in endemic populations, supports the persistence of this reservoir, impacting TB risk dynamics in multi-host systems [70,71].

This study emphasizes the importance of integrating genomic data with ecological and epidemiological information. For example, isolates from captive animals at a WCC (Limpopo) were genetically linked to free-ranging African buffalo in the GKCA, suggesting a shared source or historical transmission event. Such insights are essential for informing targeted control strategies, particularly in regions where wildlife, livestock and humans interact closely [7,12].

Despite its strengths, the study had several limitations. Sampling was opportunistic rather than part of a structured surveillance programme, leading to biases in species representation, geographic distribution and collection period. In particular, enhanced African buffalo surveillance and culling activities in 2017–2018 led to an overrepresentation of this species in the dataset. Another bias was that no *M. bovis* isolates were available from wildlife between 2002 and 2014. This was due to the absence of research and surveillance of wildlife during this period and limits any interpretation of phylogenetic changes, epidemiological links or outbreaks. At the same time, regulatory restrictions on sample transport, particularly from foot-and-mouth disease zones, further limited geographic coverage and samples from affected species could not be included during certain periods. Additionally, while culture-based methods are the standard for *M. bovis* isolation, they may not fully capture within-host diversity, although recent evidence suggests that culture bias is minimal in TB studies [72]. Sequencing of *M. bovis* isolates from South African livestock would facilitate exploration of transmission at wildlife–livestock interfaces and provide a broader foundation for understanding the epidemiology of TB in multi-host systems. Future analyses of *M. bovis* WGS from South Africa should include application of Bayesian phylogenetics, time stamping of internal nodes and use of structured coalescent methods to provide greater insights [73,76].

## Conclusion

This study provides critical insights into the genetic diversity and transmission dynamics of *M. bovis* in South African wildlife. Phylogenetic relationships between *M. bovis* isolates were observed between different host species over time and in different locations, suggesting that evolutionary changes were occurring within wildlife populations, especially since the endemically infected systems have not been subjected to culling. The findings support the use of WGS as a powerful tool for TB surveillance and highlight the need for expanded testing, improved movement controls and integrated management strategies to mitigate the spread of *M. bovis* across species and landscapes. Further studies are warranted to assess the exact mechanisms of transmission among these diverse hosts and the potential impact of TB on these systems.

## Supplementary material

10.1099/mgen.0.001646 Uncited Supplementary Material 1.

10.1099/mgen.0.001646 Uncited Supplementary Material 2.

## Abbreviations

ENA: European Nucleotide Archive
GF: private game farm
GKCA: Greater Kruger Conservation Area
HiP: Hluhluwe–iMfolozi Park
KZN: KwaZulu-Natal
MGIT: Mycobacteria Growth Indicator Tubes
MGR: Madikwe Game Reserve
MPk: Marloth Park
MST: minimum spanning tree
MTBC: *Mycobacterium tuberculosis* complex
NR: nature reserves
NVSL: National Veterinary Services Laboratories
PGR: private game reserve
RD: regions of difference
TB: tuberculosis
VNTR: variable number tandem repeat
WCC: wildlife conservation centre
WGS: whole-genome sequencing
