# Supplementary material for: Whole-genome sequencing reveals genetic diversity and transmission dynamics of Mycobacterium bovis in South African wildlife
Source: Microb Genom. 2026 Feb 10;12(2):001646. doi: 10.1099/mgen.0.001646 (PMC12909536; doi:10.1099/mgen.0.001646)
Supplement: Uncited Supplementary Material 1. [file mgen-12-01646-s001.pdf]

File S1: In-house Python script used to align consensus FASTA files from all samples before extracting variants using SNP-sites v2.5.1.

```
import os
from Bio import SeqIO
from Bio.Align import MultipleSeqAlignmentb
from Bio.Seq import Seq
from Bio.SeqRecord import SeqRecord

# List to store valid sequences
sequences = []
headers = []

# Get all .fas files in the current directory
fas_files = [file for file in os.listdir('.') if file.endswith('.fas')]

# Iterate over the .fas files
for file in fas_files:
    # Open each .fa file and read the sequence
    with open(file, 'r') as f:
        try:
            record = next(SeqIO.parse(f, 'fasta'))
            sequence = str(record.seq)
            sequences.append(sequence)
            headers.append(record.description)
        except Exception as e:
            print(f"Error processing file {file}: {e}")

# Create a MultipleSeqAlignment object
alignment = MultipleSeqAlignment([SeqRecord(Seq(seq), id=header) for seq, header in
zip(sequences, headers)])

# Save the alignment to a file with .fasta extension
output_file = 'alignment.fasta'
SeqIO.write(alignment, output_file, 'fasta')

print(f"Alignment saved as {output_file}")
```
